# Seaweed Hydrocolloid Production: An Update on Enzyme Assisted Extraction and Modification Technologies

**DOI:** 10.3390/md13063340

**Published:** 2015-05-27

**Authors:** Nanna Rhein-Knudsen, Marcel Tutor Ale, Anne S. Meyer

**Affiliations:** Center for Bioprocess Engineering, Department of Chemical and Biochemical Engineering, Technical University of Denmark (DTU), Søltofts Plads, Building 229, DK-2800 Lyngby, Denmark; E-Mails: nark@kt.dtu.dk (N.R.-K.); mta@kt.dtu.dk (M.T.A.)

**Keywords:** seaweed, carrageenan, alginate, agar, hydrocolloid, enzymatic extraction

## Abstract

Agar, alginate, and carrageenans are high-value seaweed hydrocolloids, which are used as gelation and thickening agents in different food, pharmaceutical, and biotechnological applications. The annual global production of these hydrocolloids has recently reached 100,000 tons with a gross market value just above US$ 1.1 billion. The techno-functional properties of the seaweed polysaccharides depend strictly on their unique structural make-up, notably degree and position of sulfation and presence of anhydro-bridges. Classical extraction techniques include hot alkali treatments, but recent research has shown promising results with enzymes. Current methods mainly involve use of commercially available enzyme mixtures developed for terrestrial plant material processing. Application of seaweed polysaccharide targeted enzymes allows for selective extraction at mild conditions as well as tailor-made modifications of the hydrocolloids to obtain specific functionalities. This review provides an update of the detailed structural features of κ-, ι-, λ-carrageenans, agars, and alginate, and a thorough discussion of enzyme assisted extraction and processing techniques for these hydrocolloids.

## 1. Introduction

Hydrocolloids can be defined as substances that interact with water to form colloid systems either in the form of a gel or a sol system of solubilized particles. In practice, the viscosity of the system will generally increase as a result of the interaction between the hydrocolloid and water. Hydrocolloid polysaccharides have significant importance, both technologically and economically, since they are used in the food, pharmaceutical, medicinal, and biotechnological industries due to their distinct physico-chemical properties. The currently used hydrocolloid polysaccharides are derived from plant, microbial, and seaweed sources: pectin is, for example, extracted from apple pomace and citrus peel; xanthan gum is prepared by aerobic fermentation from *Xanthomonas campestris*, and agar, alginates, and carrageenans are obtained from brown and red seaweeds. Seaweed-derived hydrocolloids currently have a global value of approximately US$ 1.1 billion, which is prospected to increase [1]. Seaweeds, thus, constitute a unique source of high-value hydrocolloid polysaccharides: agars have the highest retail price per kg (18 US$/kg), whereas carrageenans currently have the highest commercial total production (60,000 ton/year) and contribute the highest total value of US$ 626 million per year, Table 1 [1].

**Table 1:** The market for seaweed-derived hydrocolloids, agars, alginates, and carrageenans [1].

Product	Global Production (ton/year)	Retail Price (US$/kg)	Approximate Gross Market Value (US$ million/year)
Agars	10,600	18	191
Alginates	30,000	12	339
Carrageenans	60,000	10.4	626

The Asia-Pacific region dominates seaweed cultivation production, followed by countries such as, Chile, Tanzania, and Madagascar [2]. In these countries, seaweed farming has had positive socio-economic benefits on the coastal communities by improving the economic and social livelihood for the people living in the coastal areas and has reduced overfishing [3].

This review describes the chemistry, properties, and applications of the three seaweed-derived hydrocolloids, carrageenans, agar, and alginate, with a focus on novel enzyme-assisted processing techniques. Enzyme technology is a tool for targeted extractions and modifications that has recently gained increased attention in relation to preserving specific structural traits and functional properties of the target products. The use of enzymes, moreover, allows for reduction of chemicals in seaweed hydrocolloid extraction and thus holds enormous potential for creation of sustainable processing of seaweed polysaccharides.

## 2. Carrageenans

### 2.1. Common Carageenan Sources

Commercial carrageenans are extracted from the carrageenophyte red seaweed genera *Kappaphycus*, *Gigartina*, *Eucheuma*, *Chondrus*, and *Hypnea*, in which the carrageenans comprise up to 50% of the dry weight [4]. κ-Carrageenan is mostly extracted from *Kappaphycus alvarezii*, known in the trade as *Eucheuma cottonii*, while ι-carrageeman is predominantly produced from *Eucheuma denticulatum*, also known as *Eucheuma spinosum.* λ-Carrageenan is obtained from seaweeds within the *Gigartina* and *Chondrus* genera, which as sporophytic plants produce λ-carrageenan while they make a κ/ι-hybrid as gametophytic plants [4,5]. Southeast Asia and Tanzania are the main producers of seaweed derived carrageenans from *Kappaphycus alvarezii* and *Eucheuma spinosum* [6].

**Table 2:** Summary of seaweed sources, hydrocolloid carbohydrate products, chemical structures (main structural units), and applications of the seaweed derived hydrocolloids carrageenans, agars, and alginates.

Seaweed Source	Products	Main Chemical Structures	Applications	Research Conducted
*Kappaphycus alvarezii*	κ-Carrageenan	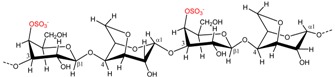	Gelling agent (stiff and brittle gel)	[7]
*Eucheuma spinosum*	ι-Carrageenan	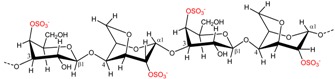	Gelling agent (flexible soft gel)	[7]
*Gigartina* spp. *Chondrus* spp.	λ-Carrageenan	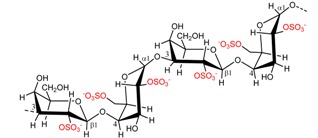	Thickener	[7]
*Kappaphycus alvarezii*	µ-Carrageenan	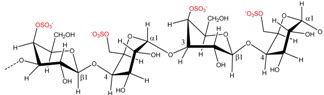	κ-Carrageenan precursor	[8]
*Eucheuma spinosum*	ν-Carrageenan	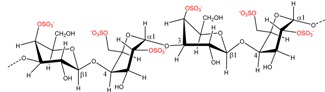	ι-Carrageenan precursor	[8]
*Gelidiella* spp. *Gelidium* spp.	Agar/Agarose	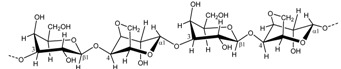	Microbiology Gelling agent (strong and rigid)	[9]
*Porphyra umbilicalis*	Porphyran	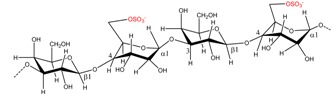	Agar precursor	[8]
*Laminaria* spp. *Sargassum* spp.	Alginate	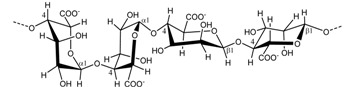	Gelling agent	[10,11]

### 2.2. Carrageenan Chemical Structure

**Figure 1:**
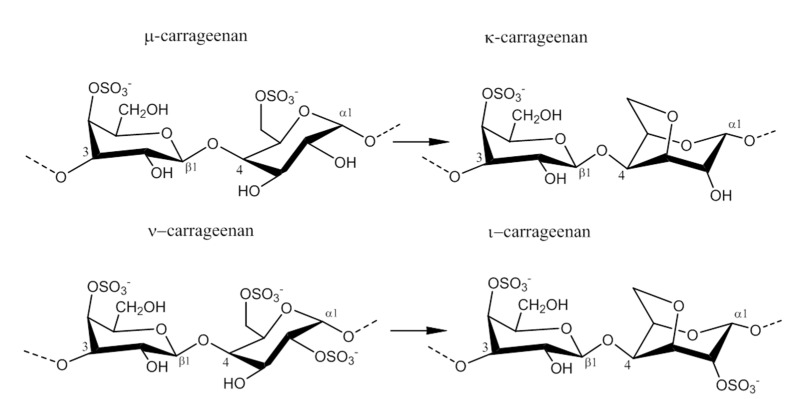
Conversion of the pre cursors μ- and ν-carrageenan into κ- and ι-carrageenan.

Carrageenans are hydrophilic sulfated linear galactans that mainly consist of d-galactopyranose units bound together with alternating α-1,3 and β-1,4 linkages. This base structure is consistent in the three main commercially used carrageenans, κ-, ι-, and λ-carrageenan, Table 2. The presence of 4-linked 3,6-anhydro-α-d-galactopyranose varies among the different carrageenans, as do the substitutions with sulfates, which are ester-linked to C2, C4, or C6 of the galactopyranose units, depending on the specific carrageenan: κ-, ι-, or λ-carrageenan. κ-Carrageenan has one sulfate ester, while ι-and λ-carrageenan contain two and three sulfates per dimer, respectively, Table 2. In addition, the galactopyranose units may also be methylated or substituted with e.g., monosaccharide residues, such as d-xylose, 4-*O*-methyl-l-galactose, and d-glucuronic acid [12,13]. Acid hydrolysis, infrared spectroscopy, and NMR analyses of commercial carrageenan typically show sulfate content of 25%–30% for κ-carrageenan, 28%–30% for ι-carrageenan, and 32%–39% for λ-carrageenan, although large differences can occur [7,14,15]. The differences in sulfate levels are explained by the fact that carrageenans are very heterogeneous carbohydrates, with structural differences coexisting within the specific type of carrageenan depending on the algal source, life-stage, and extraction method [16]. In addition, naturally occurring carrageenans contain traces of their biosynthetic precursors, μ- and ν-carrageenan, adding further to the complexity of these polysaccharides, Figure 1 [7]. Likewise, hybrid carrageenans exist, representing a mixture of the different carrageenan repeating units [5].

### 2.3. Physico-Chemical Properties of Carrageenans

Carrageenans are soluble in water, but the solubility depends on the content of hydrophilic sulfates, which lowers the solubility temperature, and the presence of potential associated cations, such as sodium, potassium, calcium, and magnesium, which promote cation-dependent aggregation between carrageenan helices [17]. Another factor affecting the physico-chemical properties in relation to viscosity and gelation is the presence of anhydro-bridges: κ- and ι-carrageenans have 3,6-anhydro-galactopyranose units, while λ-carrageenan is composed exclusively of α-1,3 galactopyranose and β-1,4 galactopyranose, Table 2.

The presence of anhydro-bridges in κ- and ι-carrageeenan is proposed to be a result of elimination of a sulfate ester present on their respective precursors, *i.e.*, in μ- and ν-carrageenan, and subsequent spontaneous anhydro-bridge formation in the desulfated monomer residue, Figure 1. The removal of the sulfate esters in µ- and ν-carrageenan reduces the hydrophilicity of the sugar residue and inverts the chair conformation from ^1^C_4_ to ^4^C_1_, Figure 1. The conformation change allows the polysaccharide to undergo conformational transitions which are conducive to the gelation properties of the anhydro-bridge containing carrageenans [8].

**Figure 2:**
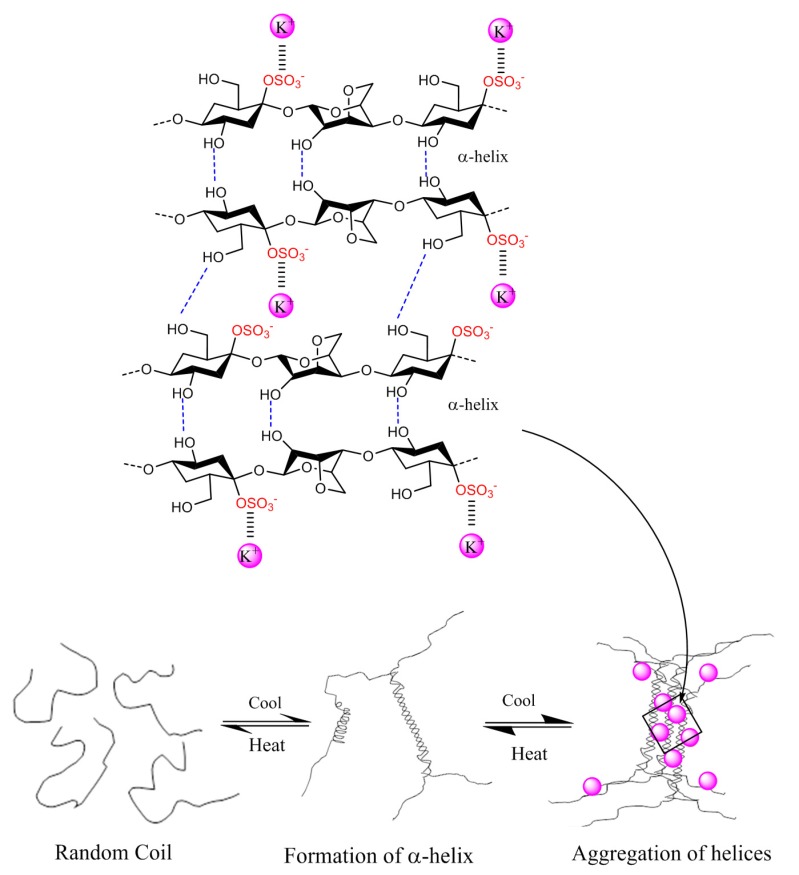
The gelation mechanism of κ-carrageenan in the presence of potassium ions.

The thermo-reversible gel formation is proposed to occur in a two-step mechanism, dependent on temperature and gel-inducing agents. At high temperatures, *i.e.*, above 75–80 °C, the carrageenans exist as random coil structures as a result of electrostatic repulsions between adjacent polymer chains. Upon cooling, the polymeric chains change conformation to helix structure. Further cooling and presence of cations (K^+^, Ca^2+^, Na^2+^) lead to aggregation of the helical dimers and formation of a stable three dimensional network, which forms through intermolecular interactions between the carrageenan chains [18,19]. The molecular details of carrageenan gelation are still uncertain. The formation of double helices prior to gelation is not fully proven, and, in principle, the formation of a duplex via chain-chain interactions may not necessarily be an unequivocal evidence for double helix formation. Nevertheless, based on the available literature data and theoretical explanations, we interpret that for the stiff κ-carrageenan gels to form, the cations, typically potassium for κ-carrageenan, function to stabilize the junction zones between the two helixes by binding to the negatively charged sulfate groups without hindering cross-linking of the two helices, Figure 2. According to this model, calcium, typically for ι-carrageenan, analogously function to cross-link the two helices through ionic salt bridges [20]. The charged sulfate esters on the other side of the monomer though, present on ι-carrageenan, encourage an extensive conformation via a repulsion effect of the negative SO_3_^−^ groups and inhibit gelation while promoting viscosity in the solution [17]. The differences in sulfate position, their proportion, and the presence of anhydro-bridges, thus, give the carrageenans distinctive gel profiles: κ-carrageenan forming strong and rigid gels, ι-carrageenan forming soft gels, and λ-carrageenan that does not gel, but still provides elevated viscosity in solution, due to a structure that does not allow helix formation, Table 2. Is has to be emphasized that natural carrageenans are heterogenous, *i.e*., have heteropolymeric structures. In practice, the rheological properties of carrageenans reflect that hybrid structures exist.

### 2.4. Enzyme Technology for Carrageenans Extraction

Carrageenans are produced as semi-refined or refined carrageenans. In the production of semi-refined carrageenans, the carrageenans are not extracted from the seaweed, but instead heated (to around 75 °C) with an alkaline solution of potassium hydroxide. The hydroxide reacts with the sulfate esters at the precursors μ- and ν-carrageenan to produce κ- and ι-carrageenan, which improves the gel strength of the product, while potassium binds to the carrageenans and promotes gel formation by preventing the hydrocolloid chains from dissolving. The seaweed containing the potassium bound carrageenan is washed, dried, and minced to powder [21]. When producing refined carrageenans, the process of semi-refined carrageenan extraction is continued further by heating (95–110 °C) the alkali treated seaweed in order to dissolve the gel matrix in the seaweed frond. The carrageenans are recovered by alcohol precipitation or gel pressing [4]. The preparation of semi-refined carrageenans is considerably cheaper than extraction of refined carrageenans, since costs associated with alcohol recovery and/or carrageenan recovery is avoided. In order to avoid the use of chemicals and the negative impacts they have on the environment, it could be of interest to process the seaweed by enzymes for the extraction of carrageenans. Apart from that, as shown for fucoidan, a non-hydrocolloid seaweed polysaccharide present in brown seaweed, the polysaccharides can also undergo degradation under severe conditions like pressure extraction, high temperatures, and high alkali concentrations [22,23].

The literature reports several examples of enzymatic extraction of carrageenans from red seaweed; Blanco-Pascual *et* *al.*, (2014) obtained a carrageenan yield of 28.65% by using an alcalase (a commercially available protease) for the extraction of a κ/ι-hybrid from *Mastocarpus stellatus*. Their product showed good gelling properties and in addition, they extracted other valuable components such as polyphenols, thereby adding value to the seaweed extraction [24]. This example emphasizes that hybrid carrageenans may be selectively extracted by use of enzymes, and that enzymes may allow for targeted production of specific gelation properties since hybrid carrageenans may exhibit unique, desirable physical properties. De Araújo *et al.*, (2012) have performed ι-carrageenan extraction experiments from *Soliera filiformis* by use of papain (a protease derived from papaya fruits)*.* Their results showed lower yield when compared to extraction by hot water (approximately 19% compared to 33%), but by enzymatic extraction, they avoided the presence of contaminant proteins, which were present when extracting by the traditional method [25]. Varadarajan *et* *al.*, (2009) have compared the use of a cellulase, *Aspergillus niger*, and traditional boiling extraction of carrageenan from *Eucheuma cottonii.* They got the highest carrageenan yield when using the cellulase Novozyme NS50013: 45% by weight compared to 37% and 37.5%, respectively. The viscosity of the cellulase-extracted carrageenan was lower than the one extracted by the traditional method though. The decrease in viscosity could be explained by the presence of impurities bound to the carrageenans as the cellulase attacks the cell walls in the seaweed to release the carrageenans and thus does not degrade the carrageenan structure itself. Likewise, the fungal treatment of the seaweed with *A. niger* resulted in the extraction of low viscosity carrageenans, most likely because the organism may have used the carrageenans as carbon source [26].

It should be added that in addition to enzymatic polysaccharide extraction from seaweed, the literature also reports aims at improving protein and metabolites extraction by enzymatic degradation: These studies have targeted enzymatic degradation of the seaweed cell wall carbohydrates simultaneously with targeted enzyme-assisted degradation of seaweed hydrocolloids. Fleurence *et al.*, (1995) thus used used κ-carrageenase and agarase in combination with cellulase for the extraction of proteins from red seaweeds. In their experiment, they showed that the highest protein yield was achieved when combining cellulase with the seaweed specific enzymes: a 10-fold increase for protein extraction from *Chondrus crispus* and a 3-fold increase from *Gracilaria verrucosa* compared to the use of cellulase alone [27]. Kulshreshtha *et al.*, used commercial carbohydrases and proteases and reported a significant improvement in extraction efficiency of bioactive materials from *Chondrus crispus* compared to aqueous extraction [28]. As stated above, the current enzymatic carrageenan extraction methods have not aimed at modifying the target polysaccharides during the extraction. However, when extracting carrageenans by enzymatic reactions, the precursors μ- and ν-carrageenan have to be converted into κ- and ι-carrageenan for achievement of purer product and better gelling abilities. Genicot-Joncour *et* *al.*, (2009) [8] have identified and purified sulfurylases that are capable of converting ν-carrageenan into ι-carrageena, Figure 1 and Table 3. Likewise, sulfurylases responsible for catalyzing the sulfate removal causing the conversion of μ-carrageenan into κ-carrageenan, Figure 1 and Table 3, have been identified [8].

Intensive research has been conducted on the hydrolysis of carrageenans and by far the most studied microorganism in respect to this is the marine bacterium *Pseudoalteromonas carrageenovora*—and the enzymes produced by this organism. From this bacterium, Potin *et* *al.*, (1995) purified and analyzed a κ-carrageenase (EC 3.2.1.83) responsible for cleavage of the β-1,4 linkages, belonging to the glycoside hydrolase (GH) 16 family, along with several β-agarases responsible for the degradation of agarose, Table 3 [29]. In 2000, Barbeyron *et* *al.*, purified a ι-carrageenase (EC 3.2.1.157) from *Zolbellia galactanivorans*—the enzyme belongs to GH family 82 along with other reported ι-carrageenase, Table 3 [30]. In 2007, Guibet *et* *al.*, isolated yet another carrageenase from *P. carrageenovora*, but this enzyme acts only on λ-carrageenan, Table 3 (EC 3.2.1.162). Comparisons of sequences, catalytic sites, and mechanisms have revealed that this latter enzyme belongs to another family of glycoside hydrolases, a new family yet to be specified [31].

Digestion by carrageenases generates oligo-galactans of various sizes, most likely carbohydrates with a degree of polymerization (DP) of 2, 4, and 6. The reason for the production of different DPs is a result of the heterogenous carrageenan structure and the mechanisms that the enzymes follow. The alternating α-1,3 and β-1,4 linkages in the carrageenans results in successive β-1,4 linkages to be in opposite orientations and hence only every second disaccharide is in the right position for cleavage [30,32]. The three carrageenases all have an endo-lytic mode of action, in which they act on linkages in the middle of the chains, resulting in the formation of DP6s [31,33,34]. The main products from κ- and ι-carrageenase digestion are DP4s and DP2s, indicating a processive mechanism, in which the enzyme does not dissociate from the substrate and instead slides along the polysaccharide, cleaving all possible bonds. The tunnel-shaped active sites, found in both κ- and ι-carrageenases, further indicate a processive mechanism, where the substrate is enclosed in the active site of the enzyme. This processive behavior favors the formation of DP4s and DP2s [30,33,35]. λ-Carrageenase on the other hand, proceeds in a more random manner, resulting in higher amounts of DP6s (and possible other higher DPs as products) compared to the products from κ- and ι-carrageenase hydrolysis. Enzymes responsible for the conversion of smaller carrageenan oligosaccharides have, to our knowledge, only been reported for κ-carrageenan DP4, which is converted into κ-carrageenan DP2 by a carratetraose 4-*O* monosulfate β-hydrolase, Table 3 [36]. However, some studies indicate that the carrageenases can attack the last β-1,3 linkages for the formation of monosaccharides with prolonged incubation time [31,32,34]. Several carrageenases have been identified so far, which all degrade carrageenan substrates, but differ in their substrate specificity, mechanism, processivity, structure, sequence, and enzyme family. The molecular mechanism for hydrolysis of the β-1,3 bonds differs between the different carrageenases. Hence, κ-carrageenases retain the anomeric configuration, while ι- and λ-carrageenases invert the anomeric [29,34]. From the strict substrate specificity it seems that carrageenases recognize the sulfation pattern, which indicates that cleavage of the internal β-1,4 linkages is the first step in the degradation of carrageenans.

Desulfation of carrageenans causes them to lose their gelling properties and is thus a less studied area, when their main application is exactly due to these qualities. Nevertheless, McLean and Williamson (1979) have identified a sulfatase from *P. carrageenovora* capable of removing the sulfate group on κ-carrageenan oligosaccharides, Table 3 [37]. An ι-carrageenan sulfatase removing the sulfate ester at position 4 in ι-carrageenan has only been identified recently from a *Pseudomonas* sp. [38]. This enzyme does not act on the sulfate at position 4 in κ-carrageenan or the sulfate at position 2 in ι-carrageenan, indicating that it specifically recognizes the sulfate on 3,6-anhydro-d-galactopyranoses [38]. These results indicate that the sulfatases are highly specific, as is the case for the carrageenases, but with limited knowledge about the topic, a great deal of research is still required to fully understand and control enzyme catalyzed desulfation of carrageenans. Research on other polysaccharide-acting sulfatases supports the assumption on substrate specificity: As an example, the 2S-heparan sulfatase from *Flavobacterium heparinum* is inactive on 6S-heparan sulfates and reciprocally the 6S-heparan sulfatase does not recognize 2S-heparan sulfates [39].

### 2.5. Carrageenans Applications

Due to the physico-chemical properties of carrageenans, they are often used as stabilizers, gelling agents, emulsifiers, and thickeners in the food and baking industries (ice-cream, cheese, jam, bread dough). Other applications include their use as binders in toothpaste, thickeners and stabilizers in cosmetics, and as smoothers in pet food. The semi-refined carrageenan flour is colored, and may have a high bacterial count, and is thus not appropriate for human consumption but is used in canned pet food, where the canning process destroys any living organisms [40]. More recently, carrageenans have attracted attention in the pharmaceutical industry, since it has been shown, that carrageenan can inhibit attachment of viruses such as the human papillomavirus, dengue virus, and herpes virus. In addition, carrageenans are used in several drug delivery systems as matrixes to control drug release, microcapsules, and microspheres [41].

**Table 3:** Summary of enzymes reported in relation to modification of carrageenans, agar, and alginate.

Hydrocolloids	Enzymes	Organisms	Catalytic Reaction	Research Conducted
**κ-Carrageenan**	κ-Carrageenase EC 3.2.1.83 GH 16	*Pseudoalteromonas carrageenovora*	Endohydrolysis of (1,4)-β-d-linkages between d-galactose 4-sulfate and 3,6-anhydro-d-galactose	[29]
**κ-Carrageenan**	Sulfatase	*Pseudomonas carrageenovora*	Eliminates sulfate from d-galactose 4-sulfate, producing d-galactose	[37]
**κ-Carrageenan**	Carratetraose-4-*O* monosulfate-β-hydrolase	*Pseudomonas carrageenovora*	Hydrolysis of (1,4)-β-d-linkages between between d-galactose 4-sulfate and 3,6-anhydro-d-galactose in κ-carrageenan DP4	[36]
**κ-Carrageenan**	Sulfurylase I and II	*Chondrus crispus*	Eliminates sulfate from d-galactose 6-sulfate of μ-carrageenan, producing 3,6 anhydro-d-galactose residues	[42]
**ι-Carrageenan**	ι-Carrageenase EC 3.2.1.157 GH 82	*Zolbellia galacta*	Endohydrolysis of (1,4)-β-d-linkages between d-galactose 4-sulfate and 3,6-anhydro-d-galactose-2-sulfate	[30]
**ι-Carrageenan**	Sulfatase	*Pseudoalteromonas atlantica*	Eliminates sulfate from d-galactose 4-sulfate, producing d-galactose	[38]
**ι-Carrageenan**	Sulfurylases I and II	*Chondrus crispus*	Eliminates sulfate from d-galactose 6-sulfate of ν-carrageenan, producing 3,6 anhydro-d-galactose residues	[8]
**λ-Carrageenan**	λ-Carrageenase EC 3.2.1.162	*Pseudoalteromonas carrageenovora*	Endohydrolysis of (1,4)-β-d-linkages between d-galactose 2-sulfate and d-galactose 2,6-sulfate	[31]
**Agar**	Gal-6-sulfurylase EC 2.5.1.5	*Porphyra umbilicalis*	Eliminates sulfate from l-galactose 6-sulfate of porphyran, producing 3,6-l-anhydrogalactose	[43]
**Agar**	α-Agarase EC 3.2.1.158	*Thalassomonas agarivorans* JAMP-A33	Endohydrolysis of (1,3)-α-l-linkages between d-galactose and 3,6-anhydro-l-galactose	[44]
**Agar**	β-Agarase EC 3.2.1.81	*Alteromonas* sp. SY37-12	Hydrolysis of (1,4)-β-d-linkages between 3,6-anhydro-l-galactose and d-galactose in agar	[45]
**Alginate**	Mannuronate lyase EC 4.2.2.3 PL5	*Azotobacter chroococcum*	Cleavage of polysaccharides with β-d-mannuronate	[46]
**Alginate**	Guluronate lyase EC 4.2.2.11 PL7	*Klebsiella aerogenes*	Cleavage of polysaccharides containing α-l-guluronate	[47]
**Alginate**	Mannuronan C5 epimerase	*Azotobacter vinelandii*	Epimerisation of β-d-mannuronic acid residues at C5	[48]

## 3. Agars

### 3.1. Common Red Seaweed Sources

Agars are industrially produced from the agarophytes red seaweed genera *Gelidium*, *Gracilaria*, and *Gelidiella* [2]. *Gelidium* seaweed is harvested in large quantities on the north coast of Spain, at the southern coast of Portugal, and at the west coast of Morocco. *Gracilaria* species are widely distributed in colder waters such as southern Chile and the Atlantic coast of Canada, with some species adapted to tropical waters, e.g., around Indonesia. Commercial cultivation of *Gracilaria* was established using *Gracilaria chilensis*, which is a native red seaweed species originating from the southern coast of Chile. Significant quantities of *Gracilaria* sp. is now cultivated in ponds and estuaries in Asia, notably in China, in the southern provinces of Guangxi and Hainan, and also in Indonesia, and Vietnam, whereas *Gelidiella acerosa* is the main source of agar in India [2] The global production of agar was approximately 10,600 ton/year, with an estimated worth of US$ ~191 million in 2014, Table 1.

### 3.2. Chemical Structure of Agar

Like carrageenans, agars are hydrophilic galactans consisting of galactopyranose units with alternating α-1,3 and β-1,4 linkages, but, whereas the α-linked galactopyranose is in the d-configuration in carrageenans, agar is made up of l-galactopyranose units. Some agars contain traces of its precursor porphyran: d-galactose and l-galactopyranose 6-sulfate [12]. The presence of 3,6-anhydro-l-galactopyranose was first proposed by Rees (1961) [49] via enzymatically synthesized 3,6-anhydro-l-galactopyranose with porphyran from l-galactose 6-sulfate units. Later on, various substitutions in which the most frequent are methylated galactose units such as 6-*O*-methyl-d-galactose and 4-*O*-methyl-l-galactose, l-galactose, methyl-pentose, and xylose were described for agar by Araki *et al*., (1967) [50]. Agar extracted from the red seaweed *Laurencia pinnatifida* Lamour was identified to contain 2-*O*-methyl-3,6-anhydrogalactose, 2-*O*-methyl-l-galactose 6-sulfate, and d-galactose 2-sulfate [51]. The 2-*O*-methylated anhydro-sugar has now been confirmed to be the major sugar in agar from *Gracilaria eucheumoides* Harvey, where it coexists with 6-*O*-methyl-d-galactose and galactose 4-sulfate [14,52]. Craigie and Jurgens (1989) established that 4-*O*-methyl-l-galactose occurs as a branch on galactose in the polymer backbone. Methylated agar is found mostly on the commercial agarose which contain some 6-*O*- and/or 2-*O*-methylated repeating units [53].

Agarose refers to the neutral unmodified backbone of agar, of which around 20% of the dimers carry methyl or sulfate groups, while agaropectin is the modified part of agar [19]. The complexity of the agar structure is a challenge in relation to establishing a standard processing technology for agar. Nevertheless, most of the natural chemical modifications, except the biological precursor, do not affect the helical conformation of the agar polysaccharides, but they may have an effect on aggregation of helices and as a consequence affect the gelation properties [54].

### 3.3. Physico-Chemical Properties of Agar

The gelling and solubility properties of agar polysaccharides are outstanding among the hydrocolloid polysaccharides because of their relative hydrophobicity: The basic structure is made up of repeating units of alternating 1,3-linked β-d-galactopyranose and 1,4-linked 3,6-anhydro-α-l-galactopyranose that allows agar to form helical dimers according to a mechanism similar to that of the carrageenans described above (Section 2.3). When 3,6-anhydrogalactose is replaced by its biological precursors, l-galactose 6-sulfate or l-galactose, helix formation and gel formation is partially prevented because of “kinks”, *i.e.*, the helix has breaking units that lack the 3,6-anhydride bridge [49].

A comparison of the physico-chemical properties of agar and carrageenan (presumably κ-carrageenan) shows that the gel strength of agar is 2–10 times higher than that of carrageenan, and that the melting point of agar is close to the boiling point of water, whereas the melting point of a carrageenan gel is 50–70 °C, Table 4. The increased gel strength and the higher melting point of agar gels are believed to be associated with the lower content of the anionic sulfates. However, the viscosity of agar in solution at 60 °C is lower than that of carrageenan, Table 4. The difference is due to the lower molar mass of the agar polysaccharides as compared to carrageenan, for commercial agar preparations, the average molecular weight typically ranges from 36 kDa to 144 kDa; in contrast, the solubility of agar depends on the ability of the solvent to disrupt and melt the ordered conformations, not the molecular weight [55].

In addition, high concentration of methoxyl and 3,6-anhydrogalactose in agar increases its hydrophobic properties, allowing agar solubility in hot solutions of 40%–80% aqueous ethanol [52]. The physico-chemical properties makes agar gels strong and rigid [56], but as for carrageenans, the natural products are hybrid heteropolymers and may harbor different heteropolymeric subunits.

**Table 4:** Physico-chemical properties for agar and carrageenans. The numbers are estimates. Viscosity values are given as (centipoise, cP) that is equivalent to N·s·m^−2^ [56].

Properties	Agar	Carrageenan
Solubility	Boiling water	Boiling water
Gel Strength (1.5% at 20 °C)	700–1000 g/cm^3^	100–350 g/cm^3^
Viscosity (1.5% at 60 °C)	10–100 centipoise	30–300 centipoise
Melting point	85–95 °C	50–70 °C
Gelling point	32–45 °C	30–50 °C

### 3.4. Extraction and Processing of Agar

The extraction procedure for agar is dependent on the specific seaweed species, but generally consists of an alkali treatment followed by hot-water extraction. As described above for carrageenans, the alkali treatment causes a chemical change in agar (formation of the 3,6-anhydro-galactopyranose) resulting in increased gel strength. The hot-water extraction is done at temperatures around 100 °C for around 2–4 h, sometimes under pressure. The agar dissolves in the water, seaweed residuals are removed by filtration, and the agar is recovered by alcohol precipitation [41]. Agarose preparation is done by fractional precipitation methods with e.g. polyethylene glycol 6000 [42], adsorption methods with e.g., aluminum hydroxide [43], or chromatography methods such as ion-exchange chromatography [44].

For extraction of agar there is a need for mild extraction conditions that can promote solubility and gel strength and avoid harmful effects on the environment and destruction of the valuable carbohydrates. As is the case for carrageenans, the anhydrogalactose accounts for the gelling capacities of agar, thus the precursor porphyran having l-galactose 6-sulfate has to be converted into 3,6-anhydrogalactose. The synthesis of 3,6-anhydro-l-galactose has been carried out using a Gal-6-sulfurylase whose activities have been demonstrated by Rees (1961) [49]. When incubating the enzyme (0.2%) and substrate (porphyran, 1%, w/v; 10 mL.) at 35 °C, the reaction leading to the formation of 3,6-anhydrogalactose, by liberation of sulfate from the ester linkages of porphyran, occurs. The detailed mechanisms of this “double reaction” desulfation and 3,6-anhydrogalactose formation is not yet fully elucidated, since 3,6-anhydrogalactose is usually combined in polysaccharides through position 4 and in a linkage. It is likely that the l-galactose 6-sulfate precursor units are similarly linked. The de-esterification of the l-galactose 6-sulfate residues, which are known to be present in porphyran, could proceed simultaneously with 3,6-anhydro-l-galactose formation, since an analogous chemical reaction is known [57].

No attempts on enzymatic extraction of agar from red seaweed have been reported, but enzymatic hydrolysis of agars has been demonstrated several times. This hydrolysis requires agarases, which are classified according to their mode of action: β-agarases that catalyze hydrolysis of the β-1,4 linkages and α-agarases that catalyze hydrolysis of the α-1,3 linkages, Table 3 [30]. The enzyme α-agarase (EC 3.2.1.158) from *Thalassomonas* sp. can use agarose, agarohexaose and neo-agarohexaose as substrates. The products of agarohexaose hydrolysis are dimers and tetramers, with agarotetraose being the predominant product, whereas hydrolysis of neo-agarohexaose gives rise to two types of trimer. While this enzyme can also hydrolyse the highly sulfated agarose porphyran very efficiently, it cannot hydrolyse the related compounds κ-carrageenan (see EC 3.2.1.83) and ι-carrageenan (see EC 3.2.1.157) [30]. The agarose 4-glycanohydrolase (*i.e.*, β-agarase, EC 3.2.1.18) catalyzes the cleavage of the β-(1→4) linkages in agarose in a random manner with retention of the anomeric-bond configuration, producing β-anomers that progressively give rise to α-anomers when mutarotation takes place [6]. The end products of the hydrolysis are neo-agarotetraose and neo-agarohexaose in the case of AgaA (β-agarase genes A), from the marine bacterium *Zobellia galactanivorans*, and neo-agarotetraose and neo-agarobiose in the case of (AgaB β-agarase gene B) [58].

### 3.5. Commercial Applications of Agar

Due to its physiochemical properties, agar is used in the food industry as a gelling agent in, e.g., ice-cream and jam, in cosmetics as, e.g., a thickener in creams, and in pharmaceuticals as, e.g., an excipient in pills [56]. In addition, agar is widely used in growth media for culturing bacteria for scientific research. Agarose is also used in biotechnological applications, notably in gel electrophoresis and agarose-based chromatography. The reason for using agarose and not agar lies in the fact that agaropectin holds unsaturated chemical bonds in the sulfate and pyruvate substitutions that bestow high UV absorption in agarose gels and interfere with the detection of nucleic acids after electrophoresis [9].

## 4. Alginates

### 4.1. Common Brown Seaweed Sources of Alginate

Alginates or alginic acids are distinguished from the other seaweed hydrocolloids because they are extracted from brown seaweeds. In brown seaweeds, alginate constitutes a key component of the seaweed cell walls and also appears to be present in the intercellular space matrix. Alginate therefore appears to be present in most brown seaweed species, but the amounts vary. The main species used for commercial alginate extraction are *Laminaria* spp., *Macrocystis* spp., *Ascophyllum* spp., *Sargassum* spp., and *Fucales* spp.—in these species, alginate comprises up to 40% of the dry matter [2,4,59]. *Laminaria japonica* (a.k.a. *Saccharina japonica*) is abundant in China and can compete with the western alginate producers. However, the low guluronic to mannuronic acid ratio (M:G) of *L. japonica* from China yields weakly gelling alginates (see below). This issue prompts Chinese alginate producers to import *Lessonia nigrescens* from Chile and Peru [60]. It has been postulated that *Sargassum* spp. are only used when no other brown seaweed is available because its alginate is usually borderline quality and the yields are low [2]. Nonetheless, it was shown that different species of *Sargassum* and extraction technology employed provide very different yields and quality of alginates [61]. Alginates can also be isolated from bacteria such as *Azotobacteria* and *Pseudomonas* [62], but at present bacterial alginate production is not employed commercially.

Europe, USA and Japan were the main producers of alginates 30 years ago, but the emergence of Chinese alginate producers has changed the alginate industry in the last decades. The global market value for alginates is currently estimated to be US$ 339 million/year, Table 1. The alginates market share by application has increased by 20% for food/pharmaceutical segments. The world production capacity has expanded by 25%, mainly in China, during the last decade [60] (although reliable figures from China are difficult to obtain).

### 4.2. Chemical Structure and Physico-Chemical Properties of Alginate

Alginates are linear polymers build up by the two monomeric uronic acids, β-d-mannuronic acid (M) and α-l-guluronic acid (G). The two uronic acids are arranged in an irregular blockwise pattern of varying proportions of MM, MG, and GG blocks, depending on algal source, extraction technique, and harvest time. The mannuronic acids form β-1,4 linkages, which gives the MM-blocks a linear and flexible conformation, while guluronic acid gives rise to α-1,4 linkages, and introduces a steric hindrance around the carboxyl groups, thereby providing a folded and rigid structure that ensures the stiffness in the polymer chain [59].

Like the other seaweed-derived hydrocolloids described in this paper, alginate has gel-formation capacities as well. In the presence of divalent cations, mostly Ca^2+^, the ions can bind to the carboxyl groups in alginate and act as cross-linkers that stabilize the alginate chains by formation of a gel-network. As shown by Grant *et* *al.* (1973), the gelation process predominantly involves cooperative binding of the divalent ions across the GG-blocks of aligned alginate chains, hence the M:G ratio has a major impact on the physico-chemical properties of alginate: Alginates with low M:G ratios (*i.e.*, having relatively high numbers of guluronic acid residues) generally form dense and brittle gels, whereas alginates with high M:G ratios (*i.e*., with a relatively low number of guluronic acid residues) produce more elastic gels [10,11].

The M:G ratio varies amongst brown seaweed taxonomic ranks (*i.e.*, order); typically *Ascophyllum nodosum* (*Fucales*) have alginates with an M:G ratio of approximately 1.2; whereas *Laminaria japonica* (*Laminariales*) have higher M:G ratios of approximately 2.2, while many *Sargassum* (*Fucales*) alginates have M:G ratios ranging from 0.8 to 1.5 [61].

### 4.3. Alginates Extraction and Processing

Alginates are extracted in different ways depending on the application, but the most commonly used procedure is the one described by Calumpong *et* *al.* (1999), which relies on extracting the alginate as sodium alginate. The method is based on converting the insoluble calcium- and magnesium-alginates present within the brown seaweed cell walls to soluble sodium alginates that are subsequently recovered as alginic acid or calcium alginate. This conversion is done by sequential addition of acid, alcohol, and sodium carbonate [63]. The extraction techniques available for alginate extraction face some difficulties in, e.g., relation to separation of the seaweed residuals that do not dissolve. As the alginate dissolves as sodium alginate, the thickness of the solution hinders filtration and the solution has to be diluted with large quantities of water. As the seaweed residuals are very fine and can clog the filter, filter aids must be provided making the process expensive. In addition, the chemicals used for extraction are believed to influence the physico-chemical properties of alginates [64]. To avoid the difficulties encountered in the traditional extraction techniques and the destructive effects they have on the functional properties there is a need for alternative extraction and processing techniques.

Enzymatic hydrolysis of alginates has been intensively studied and both β-d-mannuronate and α-l-guluronate lyases that catalyze the degradation of alginate have been isolated from marine algae, marine mollusks, and a wide range of microorganisms, Table 3 [46,47].

The two alginate lyases catalyze the degradation of alginate by a β-elimination mechanism targeting the 1,4 glycosidic bond connecting the two uronic acid monomers. A double bond is formed between the carbon atoms at position 4 and 5 in the uronic acid ring, from which the 1,4 glycosidic bond is eliminated, resulting in the production of a 4-deoxy-l-erythro-hex-4-enopyranosyluronic acid. Although the enzymes are classified according to their specificity, they usually have moderate to low processivity for the other epimer [65]. As mentioned above, lab scale studies have demonstrated that alginate can be synthesized by bacteria belonging to the genera *Azotobacter* and *Pseudomonas* where alginates are synthesized as mannuronan, and varying amounts of the M residues in the polymer are then epimerized to G residues by mannuronan C-5-epimerases [66]. In an early study conducted by Haug and Larsen (1971), mannuronan C-5-epimerases isolated from liquid cultures of *Azotobacter vinelandii* were examined to epimerize d-mannuronic acid residues to l-guluronic acid residues of calcium alginate prepared from brown algae. The results showed that both homopolymeric blocks of l-guluronic acid and blocks having an alternating sequence of M- and G-residues are formed by this enzymatic epimerization reaction [48]. Since the gel-forming, water-binding, and immunogenic properties of the polymer are dependent on the relative amount and sequence distribution of M and G residues, the available studies indicate that certain enzymes can be used for production of alginates with specialized properties. However, to our knowledge, there are no reports available that examine the addition of epimerase during extraction and processing of alginates.

To our knowledge, no attempts on enzymatic extraction of alginate from brown seaweed have been reported, but as previously described for red seaweeds, proteins and bioactive components have been isolated from brown seaweed by enzyme-assisted extraction techniques as well. These experiments have aimed at degrading the cell walls in order to release the desirable compounds from the seaweed cells. Hardouin *et* *al.*, (2013) have used carbohydrases and proteases for the extraction of antiviral compounds from the brown seaweed *Sargassum muticum* and showed that the yield could be increased by the use of enzymes when compared to the traditional extraction [67]. Anticoagulant compounds have been extracted from seven brown seaweed sources using five carbohydrases by Athukorala *et* *al.*, (2006) [68] and Heo *et* *al.*, (2005) used five commercial carbohydrases and proteases for the extraction of antioxidants from brown seaweed [69].

### 4.4. Common Applications for Alginates

Alginates are used in the food industry as stabilizers and thickeners in e.g., jelly, drinks, and desserts. In addition, alginates are important in the healthcare and pharmaceutical industry where they are being used as wound dressings and as matrices to encapsulate and/or release cells and medicine [70,71,72].

Alginate has also been reported as a suitable substrate for heavy-metal adsorption and several studies reason that brown seaweed therefore could be used for absorption of heavy metal. This application could be considered implemented as a strategic removal of toxic substances from wastewaters when cultivating seaweed for alginate extraction [73].

## 5. Conclusions

Seaweed is a unique source of valuable hydrocolloids that due to their functional properties have significant importance in the food, medicinal, and biotechnological industries. The traditional extraction techniques rely on the use of chemicals under harsh conditions. In order to maintain the functional properties of the valuable hydrocolloid polysaccharides and to avoid the use of chemicals, there is a need for milder and more selective extractions techniques.

Current literature mainly focuses on hydrolysis of the hydrocolloids, and several seaweed specific enzymes have been identified which degrade the hydrocolloid polysaccharides and thereby change the solubility and gel strength. A few studies have covered the use of commercial, microbially-derived cellulases and proteases, as well as combinations of the two with seaweed specific enzymes, for seaweed hydrocolloid extraction. Such enzyme mixtures have also been used for extraction of protein and other components from selected seaweed species. However, the commercial enzyme mixtures employed have generally been developed for terrestrial plant biomass processing, and not for seaweed carbohydrates, and some enzyme treatments increased the carbohydrates yield while maintaining the gelling properties and others decreased the hydrocolloid yield and interfered with the gelling abilities of the hydrocolloids. There is a need for developing better enzymes designed for seaweed polysaccharides processing, since the use of enzymes allows for reduction of chemicals in seaweed hydrocolloid extraction while allowing for tailor-made functional properties and thus holds enormous potential for creation of sustainable processing of seaweed polysaccharides.
